# A social-development model of the evolution of depressive symptoms from age 13 to 30

**DOI:** 10.1017/S0954579422001183

**Published:** 2022-12-01

**Authors:** Joseph P. Allen, Corey Pettit, Meghan A. Costello, Gabrielle L. Hunt, Jessica A. Stern

**Affiliations:** Department of Psychology, University of Virginia, Charlottesville, VA, USA

**Keywords:** adolescent, depression, friendships, longitudinal, predictions

## Abstract

This 17-year prospective study applied a social-development lens to the challenge of identifying long-term predictors of adult depressive symptoms. A diverse community sample of 171 individuals was repeatedly assessed from age 13 to age 30 using self-, parent-, and peer-report methods. As hypothesized, competence in establishing close friendships beginning in adolescence had a substantial long-term predictive relation to adult depressive symptoms at ages 27–30, even after accounting for prior depressive, anxiety, and externalizing symptoms. Intervening relationship difficulties at ages 23–26 were identified as part of pathways to depressive symptoms in the late twenties. Somewhat distinct paths by gender were also identified, but in all cases were consistent with an overall role of relationship difficulties in predicting long-term depressive symptoms. Implications both for early identification of risk as well as for potential preventive interventions are discussed.

Depression is the single greatest cause of disability worldwide, and rates of depression have been increasing significantly in recent years even prior to the onset of the pandemic (World Health Organization, 2017). The need for effective prevention strategies is clear, yet to a large extent our ability to develop such strategies will depend upon the quality of our understanding of the early roots of adult depressive symptoms. This study examined the early adolescent origins of adult depressive symptoms from the perspective of a developmental tasks approach (Waters & Sroufe, 1983). This approach suggests that struggles in managing the developmental task of learning to establish competence in close relationships in adolescence play a fundamental role in creating a long-term vulnerability to the development of depressive symptoms. This perspective was examined within a broader model that assessed the role of early struggles to establish relationship competence together with baseline levels of depressive symptoms and prior anxiety and externalizing symptoms.

In developing a model predicting the development of adult depressive symptoms, the principle of homotypic continuity suggests a starting place in the correlation between levels of depressive symptoms from adolescence to adulthood; to date, however, only very modest homotypic continuities have been observed over time. This is true for longer-term predictions from adolescence into the thirties (McLeod et al., 2016), but also for shorter-term predictions from adolescence into just the very early twenties (Gutman & Sameroff, 2004) (though more continuity is seen in the subgroup of adolescents with very high levels of depressive symptoms;Pine et al., 1999). This overall pattern of modest homotypic continuity suggests a need to look more broadly to understand the early roots of adult depressive symptoms.

In expanding the field of view outward in looking for adolescent-era predictors of adult depressive symptoms, several other forms of prior psychopathology warrant consideration. Anxiety symptoms are an obvious potential predictive factor, given that anxiety and depressive symptoms tend to develop similarly from childhood to adolescence and to co-occur during adolescence (Cummings et al., 2014; Moffitt et al., 2007). Depressive symptoms in adulthood have been predicted by childhood anxiety and related somatic complaints (Roza et al., 2003; Shanahan et al., 2015), although generally not in studies which control for baseline depressive symptoms. Significant comorbidity of anxiety and depression has also been observed in adults (Kessler et al., 2008). Externalizing behaviors in childhood and adolescence have also been repeatedly examined as potential predictors of adult depressive symptoms. Although results have not been entirely consistent, meta-analyses suggest that externalizing symptoms in childhood tend to predict depressive symptoms in adulthood, possibly reflecting a more general pattern of emotional dysregulation (Loth et al., 2014). This work is limited, however, in that most studies of predictions from childhood externalizing symptoms did not account for the possibility of co-occurring childhood depression. In addition, whether externalizing behavior in adolescence (vs. childhood) will be predictive is still unknown, given that the dramatic rise in delinquent activity in adolescence suggests that externalizing behavior may take on a different meaning during this period. Notably, at least one study has failed to find links of adolescent delinquent activity to depression in the early twenties (Wiesner & Windle, 2006).

Beyond symptomatic predictors, however, a developmental psychopathology perspective suggests that success or failure in accomplishing key social developmental tasks at a given stage may predict the likelihood of significant psychopathology emerging at future points in development (Waters & Sroufe, 1983). The ability to develop and maintain healthy close relationships is one such key task in adolescence. Neurodevelopmental research has demonstrated that adolescence is a sensitive period during which the brain is especially responsive to social experiences, particularly with peers, and social experiences during this period can shape neurocircuitry involved in the development of psychopathology (Blakemore, 2008). The ability to form high-quality social relationships in adolescence now appear closely linked to everything from concurrent and future academic functioning to long-term romantic and general life satisfaction (Allen et al., 2020; Li et al., 2011; Loeb et al., 2020; Marion et al., 2013). Conversely, poor or absent interpersonal relationships appear to be not only correlated with depressive symptoms in adolescence, but predictive of increasing levels of depressive symptoms over time within adolescence (Allen et al., 2006; Chango et al., 2012) and beyond (Allen et al., 2022; Narr et al., 2019). Similarly, within experimental paradigms, peer rejection has been linked to short-term increases in adolescent depressive symptoms (Platt et al., 2013). Finally, adult relationship quality has repeatedly been found to be predictable from the quality of adolescent-era social relationships, suggesting that failure to establish good quality relationships in adolescence does indeed carry forward (Kansky et al., 2017; Oudekerk et al., 2015; Pascuzzo et al., 2013).

The role of poor social relationship quality in depressive symptoms is also seen within adulthood. A review of more than fifty studies on the link between poor social relationship quality and depressive symptoms found strong evidence linking lack of social support to higher levels of depressive symptoms both concurrently and prospectively (Santini et al., 2015). These results are consistent with findings that the quality of interactions with romantic partners predicts changes in depressive symptoms over a multiyear period in older adulthood (Stafford et al., 2011). Conclusions in both cases are limited, however, due to the near complete reliance on self-reports of relationship quality and the significant shared method variance confounds this creates. No work to date has looked at predictions of change in symptoms within adulthood using methods other than self-reports of relationship quality.

In sum, the social relationship perspective on the development of depressive symptoms has much to commend it, including research identifying links of adolescent relationship quality to adult depressive symptoms (Allen et al., 2022; Narr et al., 2019). This perspective has not, however, been examined as part of a more comprehensive model that could assess competing pathways to depressive symptoms. For example, no study to date has been able to assess the relative roles of adolescent-era depressive, anxiety, and externalizing symptoms in predicting future depressive symptoms. Thus it is not even possible to rule out the possibility that unmeasured anxiety or externalizing symptoms may have driven both relationship difficulties and depressive symptoms in past studies, creating a spurious relation between the two. Nor has any attempt been made to assess evidence of the predicted developmental cascade from adolescent to young adult relationship difficulties to adult depressive symptoms.

The current study sought to address these limitations by assessing an integrative model of the adolescent-era predictors of adult depressive symptoms using a prospective, multimethod approach within a demographically diverse community sample of adolescents assessed repeatedly from age 13 to 30. Both symptom-based predictors as well as multiple markers of competence in social relationships beginning in early adolescence were examined. Given the episodic nature of depressive symptoms, repeated assessments were obtained and aggregated so as to identify enduring underlying propensities to experience depressive symptoms. Although the lack of long-term research in this area of necessity renders this study as somewhat exploratory, three specific hypotheses were assessed:

1. Poor adolescent close friendship competence, assessed via multiple sources, will predict depressive symptoms in young adulthood (i.e., beyond the late adolescent transition), even after accounting for adolescent-era depressive, anxious, and externalizing symptoms.
2. Poor friendship quality within adulthood will also predict relative increases in depressive symptoms from young adulthood to the late twenties.
3. Long-term links from adolescent-era friendship quality to depressive symptoms in the late twenties will be observed, including both direct and indirect links via intervening levels of young adult depressive symptoms.

Given the well-established role of gender differences in both depressive symptoms and in social relationships (Hankin et al., 1998; Nolen-Hoeksema & Hilt, 2009), gender was examined as both a predictor and a moderator of key social processes for each hypothesis.

## Method

### Participants

This report is drawn from a larger longitudinal investigation of adolescent social development in familial and peer contexts. Original participants included 184 seventh and eighth graders (86 male and 98 female) followed over a 17-year period from ages 13 to 30, along with collateral data collected from mothers and close friends of these adolescents. The sample was racially/ethnically and socioeconomically diverse: 107 adolescents (58%) identified as Caucasian, 53 (29%) as African American, 15 (8%) as of mixed race/ethnicity, and 9 (5%) as being from other minority groups. Adolescents’ parents reported a median family income in the $40,000–$59,999 range at the initial assessment.

Adolescents were initially recruited from the 7^th^ and 8^th^ grades of a public middle school drawing from suburban and urban populations in the Southeastern United States. Students and their peers were recruited via an initial mailing to all parents of students in the school along with follow-up contact efforts at school lunches. Families of adolescents who indicated they were interested in the study were contacted by telephone. If a student was identified as a close peer of a participant and agreed to participate in that capacity, they were no longer eligible to participate as primary participants, to reduce redundancies in the data. Thus, a ‘pure’ participation rate for primary participants is not readily obtained, as many of the most interested students were removed from the pool of potential primary participants (thus skewing the potential participation denominator). However, of all students eligible for participation, 63% agreed to participate as either target participants or as peers providing extensive collateral information in a 3-hr session. All participants provided informed assent/consent (depending upon whether they were an adolescent or an adult) before each interview session, and parents provided informed consent for adolescents. Initial interviews took place in private offices within a university academic building. Follow-up assessments were conducted in the same setting, or for participants’ living at a distance, were conducted either in local settings (e.g., hotel conference rooms), or via mail.

Participants were first assessed annually over a five-year period across adolescence from ages 13 to 17 [Mean age at first assessment = 13.35 (*SD* = .64), Mean age at last assessment = 17.32 (*SD* = .88)]. For the adult follow-up assessments, data were obtained from participants annually from age 24 (*M* = 23.78, *SD* = .97) to 30 (*M* = 30.19, *SD* = .96). In adolescence, participants also nominated the person they currently identified as “the peer to whom they were closest” to be included in the study. Close friends came in during a visit along with the target participant. Friends were close in age to participants (i.e., their average age differed by less than a month from target adolescents’ ages). Close friends within adolescence were specified to be same-gender friends, but the same friend need not be specified across different waves. Close friends in adolescence reported that they had known participants for an average of 4.3–5.7 years (*SD* = 3.1–3.8) across the various assessment periods; close friends in adulthood reported that they knew participants for an average of 10.3–11.2 years (*SDs* = 6.6–7.1). Data were also obtained from the adolescents’ mother [at adolescent mean ages 16.35 (*SD* = .87), and 22.80 (*SD* = .96)].

### Attrition analyses

Adult depressive symptom data were obtained from 94% of the original sample (*N* = 171). Attrition analyses comparing those participants with vs. without data on adult depressive symptoms revealed no significant differences on any predictor variables except for adolescent gender (14% attrition rate for males, vs. 1% for females). To best address any potential biases due to attrition in longitudinal analyses, FIML methods were used with analyses including all variables that were linked to future missing data (i.e., where data were not missing completely at random). Because these procedures have been found to yield the least biased estimates when all available data are used for longitudinal analyses (vs. listwise deletion of missing data) (Arbuckle, 1996), the entire original sample of 184 was utilized for these analyses. This full sample thus provides the best possible estimates of variances and covariances in measures of interest and was least likely to be biased by missing data.

### Procedure

In the initial introduction and throughout all sessions, confidentiality was assured to all study participants and adolescents were told that their parents and friends would not be informed of any of the answers they provided. Participants’ data were protected by a Confidentiality Certificate issued by the U.S. Department of Health and Human Services, which protected information from subpoena by federal, state, and local courts. Transportation and childcare were provided if necessary. Adolescent/adult participants, their parents, and peers were all paid for participation.

### Measures

#### *Depressive symptoms* (ages 13–17, 24–26, and 27–30)

were self-reported annually via the 27-item Childhood Depression Inventory (Kovacs & Beck, 1977) at ages 13–17, and the 21-item Beck Depression Inventory (Beck & Steer, 1987) at all later ages. These instruments are both well-validated and widely accepted self-report measures of depressive symptomatology (Kazdin, 1990). Items were rated on a Likert scale, summed for each year, and then aggregated across years to yield separate depressive symptom scores for the periods covered by age 13–17, age 24–26, and age 27–30. One outlier greater than 3.5 *SD* from the mean was identified in the age 24–26 scores and one in the age 27–30 scores; each was trimmed to the next highest value. Following this, levels of skewness and kurtosis were below 2 for all three measures. Internal consistency for both measures was high (Cronbach’s α’s ranged from .86 to .90).

#### *Anxiety symptoms* (ages 15–17, 24–26)

were assessed annually using the 21-item Beck Anxiety Inventory (Beck et al., 1988) from ages 15–17, and the 20-item trait anxiety scale from the State-trait anxiety inventory (Spielberger et al., 1999) for ages 24–32. Internal consistency was strong (Cronbach’s α’s across all scales ranged from .90–.94). Scores were aggregated across years 15–17 to yield a measure of adolescent anxiety symptoms and years 24–26 to yield a measure of young adult anxiety symptoms. Levels of skewness and kurtosis were below 2 for all measures.

#### *Externalizing symptoms* (ages 15–17).

Target adolescents’ named closest peer reported on adolescents’ externalizing behaviors annually using the short form of the externalizing scales from the Child Behavior Checklist (Achenbach & Edelbrock, 1981; Achenbach, 1991). The short form versions of the aggression, delinquency, and hyperactivity externalizing subscales (total 21-items) were validated using a large sample of delinquent youth where these subscales reliably predicted delinquency similarly to the full scales (Lizotte et al., 1992). Cronbach’s α for the scale of all externalizing items ranged from .85 to .89. Scores were aggregated across years to yield a single measure of adolescent externalizing symptoms. Levels of skewness and kurtosis were below 2.

#### *Close friendship competence* (friend ratings: ages 13–17; maternal rating: age 16).

Each year from age 13–17, close friends rated participants’ competence at establishing and maintaining a strong close friendship, using a version of the 4-item friendship competence scale from the Adolescent Self-Perception Profile, modified to obtain ratings of one’s friend instead of oneself (Harter, 1988). Mothers also rated their teen on the same items when their adolescent was age 16. Items focused on the extent to which teen had ‘a close friend they share secrets with,’ ‘a friend close enough to share really personal thoughts with,’ and a ‘really close friend to share things with.’ Results were averaged across the five years to produce the final scale for friend ratings. Internal consistency was good (Cronbach’s α’s ranged from .65 to .74 across years for close friend ratings and were .82 for the maternal rating).

#### *Externalizing Symptoms* (ages 24–26).

Target adolescents’ named closest peer reported on adolescents’ externalizing behaviors annually using the 34-item externalizing scale from the Adult Behavior Checklist (Achenbach & McConaughy, 2003; Achenbach & Rescorla, 2003). Cronbach’s α for the scale ranged from .90 to .93. Scores were aggregated across years to yield a single measure of young adult externalizing symptoms. Levels of skewness and kurtosis were below 2.

#### *Peer elationship qQuality* (maternal report, age 23).

Young adult peer relationship quality was assessed by mothers of participants using the Young Adult Adjustment Scale (Capaldi et al., 1992) in which participants were rated on 7 items using a 5-point scale tapping qualities such as getting along well with friends, and having a hard time finding friends (reverse-scored). Internal consistency was good (Cronbach’s α= .76).

#### *Close friendship quality* (friend report, ages 24–26).

The named closest peer of the target participant completed the peer scale of the Inventory of Parent and Peer Attachment (Armsden & Greenberg, 1987) annually to assess their perception of the overall quality of their relationship with the target participant in terms of the degree of trust, communication, and alienation (reverse-scored) in the relationship. This was a slight modification of this measure, which typically asks an individual to report on their friends more generally. The modification asked the closest peer to report on the participant specifically (e.g., instead of asking the close peer to respond about the prompt ‘I like to get my friends’ point of view … z the close peer was asked to respond to the prompt ‘I like to get my friend’s point of view’ and was told to answer with regard to the study participant. A composite score of the peer’s perceptions of the overall quality of this relationship is obtained from 25 five-point Likert scale items. Scores were aggregated across this three-year span to yield the final measure. Cronbach’s α ranged from .91 to .92 across this period.

## Results

### Preliminary analyses

Table 1 presents means, standard deviations, and intercorrelations among measures used in the study. The relatively low correlations among the adolescent-era friendship measures suggested that each was capturing a different aspect of friendship quality; thus, these were considered individually in analyses (vs. being combined into a single construct).

### Primary analyses

Analytic plan. For all primary analyses, SAS PROC CALIS (version 9.4, SAS Institute, Cary, NC) was employed using full information maximum likelihood handling of missing data. Hierarchical linear regressions were used to examine Hypotheses 1 and 2. Participant gender and baseline family income were entered in Step 1, with depressive symptoms entered in Step 2. At Step 3 other symptom-based predictors (anxiety and externalizing symptoms) were entered. Finally, at Step 4 social relationship predictors of depressive symptoms were entered. Potential moderation via gender was examined with regard to each of the predictors considered.

For hypothesis 3, a path analytic approach was taken. All potential predictors for each path were entered into an initial model, with non-significant paths then deleted and fit indices used to assure that no significant temporally feasible paths were omitted. Gender was also examined as a potential moderator as described below.

#### Hypothesis 1.

Poor adolescent close friendship competence, assessed via multiple sources, will predict depressive symptoms in young adulthood (i.e., beyond the late adolescent transition), even after accounting for adolescent-era depressive, anxious, and externalizing symptoms.

Results presented in Step II. of Table 2 revealed significant continuity in levels of depressive symptoms from adolescence to young adulthood (ages 24–26). In Step III, anxiety symptoms also added significantly to predicting future depressive symptoms. Even after accounting for continuity and symptom-based predictions, poor close friendship competence – as assessed by either maternal report or by a close friend – was found in Step IV. to be predictive of future depressive symptoms, accounting for an additional 6.5% of the residual variance in these symptoms. However, a significant interaction was found as shown in the table, such that poor close friendship competence as reported by friends was a strong predictor of depressive symptoms for females but was not a significant predictor for males.

#### Hypothesis 2.

Poor peer relationship quality within young adulthood will also predict relative increases in depressive symptoms from young adulthood to the late twenties.

Initial results presented in Table 3 indicated significant continuity in levels of depressive symptoms from young adulthood to the late twenties, with additional increments in prediction of depressive symptoms in the late twenties from young adult anxiety symptoms. After accounting for these factors, maternal report of poor young adult peer relationship quality added a significant increment to predicting depressive symptoms in the late twenties. Gender interactions were also observed, with depressive symptoms best predicted by prior depressive symptoms for females but by prior anxiety symptoms for males. However, inclusion of these interaction terms did not alter the strength or significance of the prediction from maternal report of peer relationship quality to depressive symptoms.

#### Hypothesis 3.

Long-term links from adolescent-era friendship quality to depressive symptoms in the late twenties will be observed, including both direct links and links via intervening levels of young adult depressive symptoms and relationship difficulties.

A multigroup (grouping by gender) path model was used to test Hypothesis 3, considering prediction of depressive symptoms in the late twenties from young adult measures of peer relationship quality and anxiety and externalizing symptoms as potential mediators in addition to young adult depressive symptoms. Two measures at ages 24–26 (close friendship quality as reported by close friend and externalizing symptoms) were not included in analyses given their previously observed lack of relation to later depressive symptoms. Gender moderation identified in Tables 2 and 3 was included in the model; no other significant moderators were identified.

**Figure 1.**
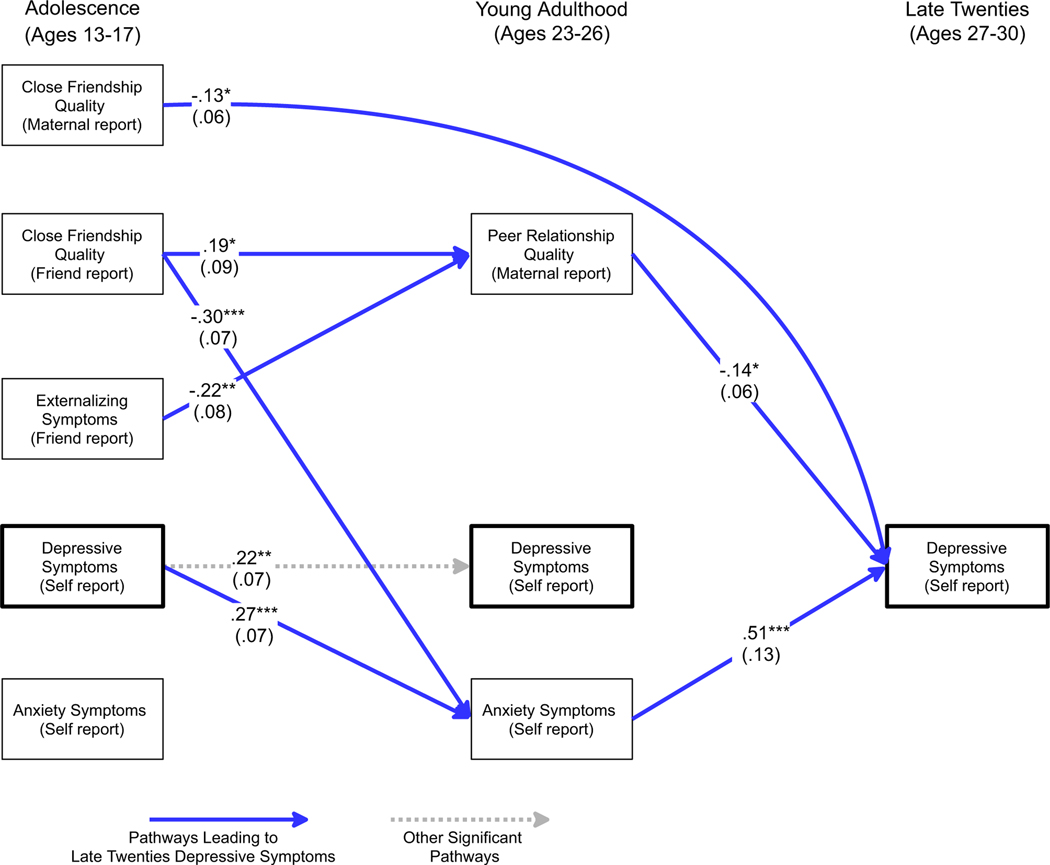
Pathways to adult depressive symptoms for males. For clarity only significant pathways are depicted and correlations among constructs collected at the same era are not shown. Standard errors are in parentheses below standardized estimates. ***p < .001. **p < .01. *p < .05.

**Figure 2.**
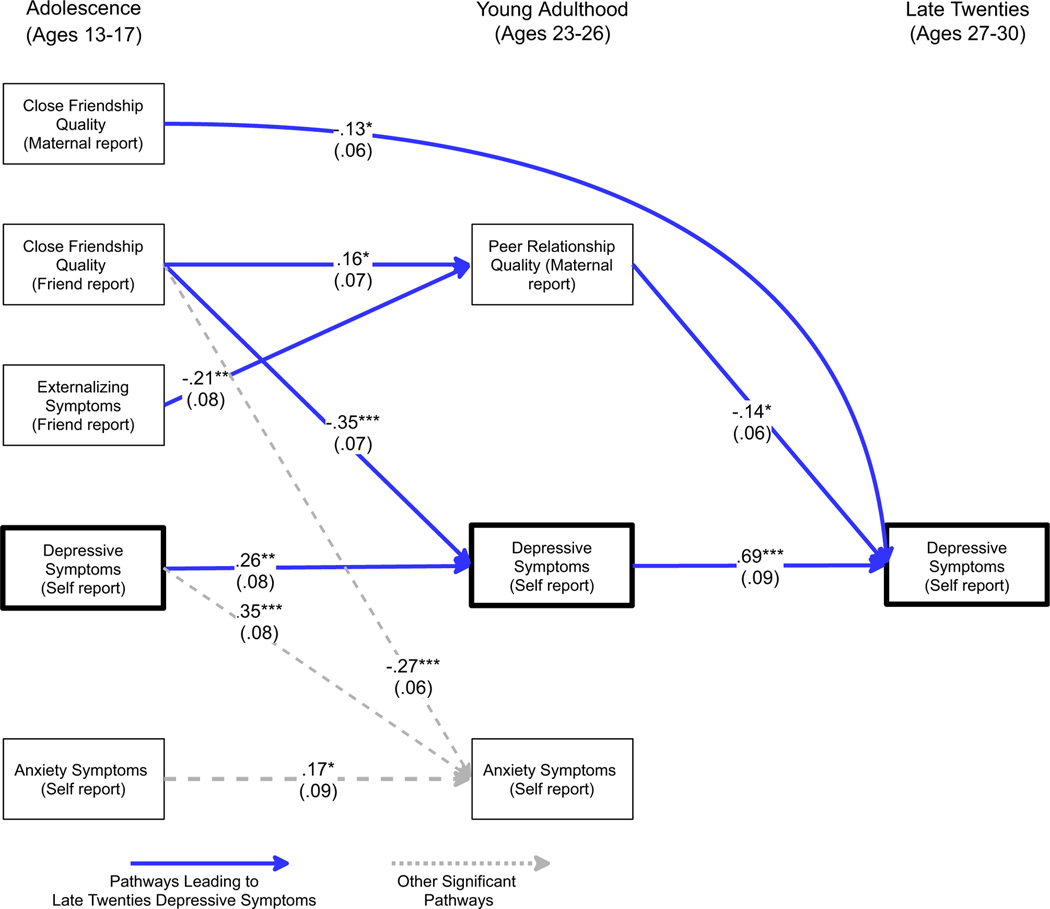
Pathways to adult depressive symptoms for females. For clarity only significant pathways are depicted and correlations among constructs collected at the same era are not shown. Standard errors are in parentheses below standardized estimates. ***p < .001. **p < .01. *p < .05.

To assess the model while considering gender moderation, two iterations of the multigroup path model were examined: the first in which all paths were constrained to be equal across gender, and the second in which paths were allowed to vary across gender where prior analyses suggested significant gender moderation. The constrained model was a poor fit to the data (*χ*^2^ (36) = 70.4, *p* < .001, GFI = .91, AGFI = .79, RMSEA = .102), whereas the second model, allowing parameters to vary across gender, fit the data well (*χ*^2^ (32) = 38.6, *p* = .19, GFI = .95, AGFI = .86, RMSEA = .047). Results are presented in Figures 1 and 2. For clarity the figures do not depict non-significant pathways nor correlations among constructs assessed contemporaneously.

As seen in both figures, across genders there was a robust pattern in which adolescent-era close friendship quality, whether measured by maternal or close friend report, was linked to future depressive symptoms. Maternal report of lower adolescent close friendship quality was directly linked to greater depressive symptoms in the late twenties, even after accounting for multiple symptom indicators at earlier time points. Similarly, as hypothesized, close-friend reports of poor adolescent-era close friendship quality predicted poorer peer relationship quality in young adulthood (maternal report), which in turn predicted greater depressive symptoms by the late twenties.

As in Table 3, young adult anxiety was a strong predictor of depressive symptoms in the late twenties for males, whereas simple homotypic continuity in depressive symptoms across this time span was observed for females. Correspondingly, for males a path was observed from early adolescent friendship quality to young adult anxiety symptoms to depressive symptoms in the late twenties. A similar path was observed for females, though this path ran from early adolescent friendship quality to young adult depressive symptoms to depressive symptoms in the late twenties.

### Post-hoc analyses

Given the findings above, as well as the potential value in assessing the pure predictive value of adolescent-era measures for long-term outcomes, a final set of analyses examined direct predictions from adolescent-era measures to depressive symptoms in the late twenties. In this analysis, no moderation by gender was observed. Results, presented in Table 4, indicate that in the full models, adolescent-era depressive symptoms drop out as a predictor, whereas externalizing symptoms and both measures of adolescent-era relationship quality significantly predicted future adult depressive symptoms. The final block of relationship quality markers accounted for 7% of the variance in adult depressive symptoms, over and above all other predictors.

## Discussion

The results of this study provide significant evidence supporting a social relationship perspective on the development of depressive symptoms from adolescence into the late twenties. The quality of adolescent-era close friendships as assessed via multiple reporters was found to be predictive of relative increases in depressive symptoms from adolescence up through age 30, even while accounting for baseline and intervening levels of anxiety and externalizing symptoms. In addition, social relationship quality predicted relative increases in depressive symptoms during the transition from young adulthood to the late twenties.

Several different pathways were observed leading from adolescent friendship quality to adult depressive symptoms. Maternal reports of adolescent close friendship quality directly predicted depressive symptoms in the late twenties, even after accounting for prior levels of depressive, anxiety, and externalizing symptoms in adolescence and young adulthood. An indirect path to adult depressive symptoms was also observed from friend-reported poor adolescent friendship quality to maternal reports of young adult peer relationship quality to depressive symptoms in the late twenties.

In addition, although the overall pattern of relationship factors predicting depressive symptoms was seen strongly for both males and females, several gender-specific pathways to adult depressive symptoms were also identified. For females, close friendship quality as reported by the friend was strongly related to relative increases in depressive symptoms from adolescence to young adulthood, and young adult depressive symptoms displayed strong continuity with depressive symptoms in the late twenties. For males, close friendship quality as reported by the friend also played a central role, but in this case it predicted relative increases in *anxiety* symptoms by young adulthood, and it was these symptoms (not young adult depressive symptoms) that best predicted depressive symptoms in the late twenties. Notably, however, this divergence in pathways across genders was not predicted *a priori* and thus must be viewed in this light.

These findings were closely linked to the different patterns across gender of continuity in anxiety and depressive symptoms from young adulthood into the late twenties. For females, close friendship quality in adolescence was directly linked to depressive symptoms in young adulthood even after accounting for prior depressive symptoms. In turn, young adult depressive symptoms strongly predicted adult depressive symptoms in the late twenties. For males, a similar pathway existed but it ran through young adult anxiety symptoms, not depressive symptoms. Although regression and path analyses entering both types of symptoms simultaneously make these findings particularly stark (relative to simple correlations, where they still appear, albeit more modestly), formal interaction tests make quite clear that these differences are robust. One explanation is that for females, difficulties in close relationships become central at an earlier age (Lempers & Clark-Lempers, 1993; Rose & Asher, 2017) and by young adulthood, these difficulties appear longstanding and lead to feelings of inferiority, helplessness and depressive symptoms; whereas for males poor relationships may lead to anxiety but not yet to a sense of helplessness and depression in young adulthood. Alternatively, it may be that in young adulthood, males are simply less willing to admit depressive symptoms, but are willing to admit to the presence of anxiety symptoms – a pattern which may then change as they age.

Notably, the predictive measures used in adolescence focused exclusively on close friendship quality. This may be important, given that prior research has found that broader peer popularity does not have the same links as close friendship quality to longer-term mental health (Narr et al., 2019). Adolescent close friendship quality was assessed from the perspective of a close friend’s report and a maternal report about the teen’s overall competence in close friendships. The specific measure used in adolescence, based on a revision to the Adolescent Self-Perception Profile (Harter, 1988), was focused more on the perceived quality of the participant’s close friendships then on broader social skills. Other research on this measure has noted that this close friendship competence scale actually is fairly tightly focused on the degree of intimacy in the friendship (Allen et al., 2022). Assessments by these two reporters were not significantly interrelated, suggesting that they each captured a different aspect of close friendship quality. One possibility is that parents focus more on their sense of whether the adolescent *has* a close friend and appears satisfied with their social life, whereas the friend measure focuses more on the extent to which the friendship is actually characterized by intimacy and closeness. Most importantly, neither of these measures relied upon adolescent self-reports, thus eliminating the methods confounds that have plagued much prior research in this area. In addition, although it is possible that parent reports were distorted by their sense of the quality of their own relationship with their offspring, this possibility is lessened by findings from other research with this sample indicating that parent-adolescent relationship quality is not a long-term predictor of offspring depressive symptoms (Allen et al., 2022).

This study also extends prior results in part by showing that predictions from relationship qualities are found even after accounting for predictions from symptoms other than just depressive symptoms. This makes it less likely that comorbid symptoms, such as anxiety, could have driven both relationship difficulties and future depressive symptoms, thus addressing a key limitation of even some of the methodologically strongest longitudinal studies to date (Allen et al., 2022; Narr et al., 2019). This is also the longest span across which relationship difficulties have been linked to depressive symptoms using relationship measures not dependent upon self-reports. In addition, this is the first study of which we are aware that examined friendship quality assessed via other than self-report as a predictor of relative change in depressive symptoms from young adulthood onward. Taken together, these findings provide the most robust evidence to date of the existence of a substantial long-term link from poor adolescent social relationships to adult depressive symptoms. From a developmental psychopathology perspective, such heterotypic continuity is not surprising. Predictions from peer-rated close friendship quality to lower levels of adult depressive symptoms are consistent with research highlighting the role of problematic social relationships in adult depressive symptomatology (Santini et al., 2015) as well as with evidence that addressing relationship difficulties within adulthood can reduce symptoms (Cuijpers et al., 2011; Weissman et al., 2017).

Although even lagged longitudinal research cannot establish causal links, what the present findings can do without qualification is suggest a promising avenue for risk assessment. Lack of competence in establishing and maintaining close friendships in adolescence appears identifiable by external parties (e.g., friends and mothers) and potentially actionable by interventions which may enhance adolescent capacities for social connection and which have been shown to reduce depressive symptoms (Allen et al., 2021; Costello et al., 2022). Notably, in direct predictive models, poor friendship competence predicted adult depressive symptoms, whereas concurrent adolescent depressive symptoms did not. This further suggests the importance of attending to key developmental tasks in predicting future psychopathology, rather than relying simply upon symptom reports. Given growing evidence regarding the long-term links of adolescent friendship quality to other physical and mental health outcomes (Allen et al., 2015, 2018, 2022), this appears a promising area for future research and intervention efforts to pursue, including for teens who are not currently depressed but may be at risk for future depressive symptoms given an absence of good close friendships.

In addition to the primary findings of the study, the comprehensive model tested also yielded several other findings of note. First, this was one of the first studies to assess the capacity of adolescent externalizing symptoms to predict adult depressive symptoms while also accounting for concurrent depressive symptoms in adolescence. Adolescent externalizing symptoms were assessed via close friend reports, thus eliminating methodological confounds as an explanation for findings. Findings suggested a modest relation of externalizing symptoms to relative increases in depressive symptoms from adolescence to young adulthood. These findings provide further support for recognizing likely heterotypic continuity in symptom profiles over time, with externalizing symptoms appearing as a significant direct predictor of later depressive symptoms in multivariate models in which concurrent depressive symptoms did not serve as a predictor. Findings also provided evidence of an indirect pathway from externalizing symptoms in adolescence to problematic close friendships in young adulthood to future depressive symptoms. One explanation for both findings is that adolescent externalizing symptoms may reflect an underlying developmental difficulty conforming behavior to larger social norms – a difficulty which may lead to future relationship difficulties and depressive symptoms. Alternatively, it is also possible that adolescent externalizing behavior is actually a means of covering up or distracting from underlying depressive symptoms which then display continuity across time.

There were also several limitations of note regarding the findings presented. Most importantly, even a long-term lagged longitudinal study is not sufficient to establish causal relations among constructs. It may well be, for example, that other unmeasured dispositional or situational factors were driving both adolescent relational difficulties and future depressive symptoms. In addition, given the relatively small sample size, the weightings of individual parameters and the presence vs. absence of specific pathways to adult depressive symptoms are all subject to considerable variability. Although the overall conclusion that relationship difficulties are strongly linked across time to depressive symptoms appears quite robust, much work remains to be done to specify the precise mechanisms and pathways that can best characterize the nature of this linkage. It is also important to note that this was a community not a clinical sample and that the focus of the study was upon depressive symptoms rather than diagnosed major depression. Although it is now well-established that sub-clinical levels of depressive symptoms are associated with quite significant levels of distress and dysfunction (Ingram & Siegle, 2002; Lewinsohn et al., 2000), further research is needed to assess the generalizability of these findings to more severely disturbed populations. Finally, although one intervening time point at ages 23–26 was examined, it may be that other slightly earlier time would be more sensitive to developmental transitions occurring between adolescence and adulthood.

Given these limitations, what is nevertheless clear is that the potential mental health significance of relationship difficulties within adolescence extends well beyond the adolescent years. The extension of predictions from multiple reports of adolescent friendship quality to symptoms at ages 27–30, and the identification of intervening symptom levels and relationship markers significantly deepens our understanding of the role of relationship quality in the development of depressive symptoms across a substantial early portion of the lifespan. Prior research has suggested that adolescent close friendships may well function as a critical zone of proximal social development in the process of learning to manage a broad range of future adult social challenges (Allen et al., 2022). At a minimum, the current findings suggest the value in paying greater attention to adolescent social relationships as we seek to understand the developmental pathways leading to (or allowing us to prevent) the development of depressive symptoms in adulthood.

## Figures and Tables

**Table 1.** Correlations among primary constructs

	Mean	*SD*	Range	2	3	4	5	6	7	8	9	10	11
1. Gender (1 = M, 2 = F)	-	-		.06	.21**	−.00	.19*	−.09	.16	.06	−.16	.21**	20**
2. Depressive symptoms (13–17)	6.22	4.32	.17–20.2	-	.54***	.08	−.10	−.20*	.34***	.44***	−.05	−.02	.26***
3. Anxiety symptoms (15–17)	6.52	6.84	0–45		-	.03	−.02	−.20*	.31***	.34***	−.10	−.03	.28***
4. Externalizing symptoms (15–17)	3.87	3.86	0–20			-	−.22	.07	.17*	.15*	−.23**	−.07	.23**
5. Close-friendship competence (close-friend report; 13–17)	4.38	0.63	2–5				-	.05	−.24**	−.30***	.12	.08	−.21*
6. Close-friendship competence (maternal report; 16)	13.1	1.45	8–18					-	−.24**	−.18*	.08	−.05	−.26**
7. Depressive symptoms (24–26)	5.28	5.01	0–22.4						-	.77***	−.17	.00	.64***
8. Anxiety symptoms (24–26)	36.5	8.95	20.3–59.7							-	−.17*	−.01	.58***
9. Peer relationship quality (maternal report, 23)	30.5	3.81	21–35								-	.13	−.31***
10. Close friendship quality (friend report, 24–26)	108.2	9.80	73.4–125									-	−.04
11. Depressive symptoms (27–30)	5.03	5.40	0–18.1										-

*Note*. Participant age(s) at time of assessment are in parentheses along with reporter source if other than self-report.

*** *p* < .001.

** *p* < .01.

* *p* < .05.

**Table 2.** Adolescent predictors of depressive symptoms at ages 24–26

	Depressive symptoms (ages 24–26)			
	
	β_entry_ (S.E.)	β_final_ (S.E.)	Δ*R^2^*	*R^2^*

**Step I.**				

Gender (M = 1, F = 2)	.16 (.07)*	.13 (.05)		

*Statistics for step*			.025*	.025*

**Step II.**				

Depressive symptoms (13–17)	.33 (.07)***	.22 (.08)*	.108***	.133***

*Statistics for step*				

**Step III.**				

Anxiety symptoms (15–17)	.14 (.08)*	.14 (.08)		

Externalizing symptoms (15–17)	.14 (.07)	.06 (.07)		

*Statistics for step*			.035*	.168***

**Step IV. Relationship predictors**				

Close-friendship competence (close-friend report; 13–17)	−.19 (.07)**	−.20 (.07)**		

Close-friendship competence (maternal report; 16)	−.16 (.08)*	−.14 (.07)		

*Statistics for step*			.070**	238***

**Step V. Gender moderation**				

Gender × externalizing symptoms (β_females_ = .19*; β_males_ = −.08)	.13+ (.07)	.13+ (.07)		

Gender × cls. frndship comp (friend rept.) (β_females_ = −.32***; β_males_ = −.13)	−.14* (.07)	−.14* (.07)		

*Statistics for step*			.041**	279***

Note. Participant age(s) at time of assessment are in parentheses along with reporter source if other than self-report.

*** *p* < .001.

** *p* < .01.

* *p* < .05.

+ *p* = .05.

**Table 3.** Young adult predictors of depressive symptoms at ages 27–30

	Depressive symptoms (ages 27–30)			
	
	β_entry_ (S.E.)	β_final_ (S.E.)	Δ*R*^2^	Total *R*^2^

**Step I.**				

Gender (M = 1, F = 2)	.20 (.07)**	.09 (.06)		

*Statistics for step*			.041**	.041**

**Step II.**				

Depressive symptoms (24–26)	.62 (.05)***	.38 (.08)***	.371***	.412***

*Statistics for step*				

**Step III.**				

Anxiety symptoms (24–26)	.22 (.09)**	.25 (.08)**		

Externalizing symptoms (24–26)	.00 (.06)	−.03 (.06)		

*Statistics for step*			.020	.432***

**Step IV. Relationship predictors**				

Peer Relationship quality (maternal report, 23)	−.20 (.06)**	−.16 (.06)*		

Close Friendship quality (friend report, 24–26)	.03 (.07)	.00 (.06)		

*Statistics for step*			.039**	.471***

**Step V. Gender moderation**				

Gender × depressive symptoms (β_females_ = .68***; β_males_= −.02)	.37 (.09)***	.36 (.09)***		

Gender × anxiety symptoms (β_females_ = .07; β_males_ = .53***)	−.28 (.09)**	−.20 (.09)*		

*Statistics for step*			.056***	.527***

Note. Participant age(s) at time of assessment are in parentheses along with reporter source if other than self-report.

*** *p* < .001.

** *p* < .01.

* *p* < .05.

+ *p* = .05.

**Table 4.** Direct predictions from adolescent predictors to depressive symptoms at ages 27–30

	Depressive symptoms (ages 27–30)			
	
	β_entry_ (S.E.)	β_final_ (S.E.)	Δ*R*^2^	Total *R*^2^

**Step I.**				

Gender (M = 1, F = 2)	.20 (.07)**	.17 (.07)*	.041*	.041*

*Statistics for step*				

Step II.				

Depressive symptoms (13–17)	.24 (.07)***	.10 (.08)	.059***	.100***

*Statistics for step*				

Step III.				

Anxiety symptoms (15–17)	.15 (.08)	.14 (.08)		

Externalizing symptoms 15–17	.21 (.07)**	.19 (.07)**	.058**	.158***

*Statistics for step*				

**Step IV. Relationship predictors**				

Close-friendship competence (close-friend report; 13–17)	−.16 (.07)*	−.18 (.07)*		

Close-friendship competence (maternal report; 16)	−.21 (.08)**	−.21 (.08)**		

*Statistics for step*			.070**	.234***

Note. Participant age(s) at time of assessment are in parentheses along with reporter source if other than self-report.

*** *p* < .001.

** *p* < .01.

* *p* < .05

+ *p* = .05. (S.E.) = standard error of the estimate.
